# P2X7 receptor activation ameliorates CA3 neuronal damage via a tumor necrosis factor-α-mediated pathway in the rat hippocampus following status epilepticus

**DOI:** 10.1186/1742-2094-8-62

**Published:** 2011-06-02

**Authors:** Ji-Eun Kim, Hea Jin Ryu, Tae-Cheon Kang

**Affiliations:** 1 Department of Anatomy & Neurobiology, Institute of Epilepsy Research, College of Medicine, Hallym University, Chunchon, Kangwon-Do 200-702, South Korea; 2 Ji-Eun Kim, Department of Neurology, UCSF, and Veterans Affairs Medical Center, San Francisco, California 94121, USA

## Abstract

**Background:**

The release of tumor necrosis factor-α (TNF-α) appears depend on the P2X7 receptor, a purinergic receptor. In the present study, we addressed the question of whether P2X7 receptor-mediated TNF-α regulation is involved in pathogenesis and outcome of status epilepticus (SE).

**Methods:**

SE was induced by pilocarpine in rats that were intracerebroventricularly infused with saline-, 2',3'-O-(4-benzoylbenzoyl)-adenosine 5'-triphosphate (BzATP), adenosine 5'-triphosphate-2',3'-dialdehyde (OxATP), A-438079, or A-740003 prior to SE induction. Thereafter, we performed Fluoro-Jade B staining and immunohistochemical studies for TNF-α and NF-κB subunit phosphorylations.

**Results:**

Following SE, P2X7 receptor agonist (BzATP) infusion increased TNF-α immunoreactivity in dentate granule cells as compared with that in saline-infused animals. In addition, TNF-α immunoreactivity was readily apparent in the mossy fibers, while TNF-α immunoreactivity in CA1-3 pyramidal cells was unaltered. However, P2X7 receptor antagonist (OxATP-, A-438079, and A-740003) infusion reduced SE-induced TNF-α expression in dentate granule cells. In the CA3 region, BzATP infusion attenuated SE-induced neuronal damage, accompanied by enhancement of p65-Ser276 and p65-Ser311 NF-κB subunit phosphorylations. In contrast, OxATP-, A-438079, and A-740003 infusions increased SE-induced neuronal death. Soluble TNF p55 receptor (sTNFp55R), and cotreatment with BzATP and sTNFp55R infusion also increased SE-induced neuronal damage in CA3 region. However, OxATP-, sTNFp55R or BzATP+sTNFp55R infusions could not exacerbate SE-induced neuronal damages in the dentate gyrus and the CA1 region, as compared to BzATP infusion.

**Conclusions:**

These findings suggest that TNF-α induction by P2X7 receptor activation may ameliorate SE-induced CA3 neuronal damage via enhancing NF-κB p65-Ser276 and p65-Ser311 phosphorylations.

## Background

Status epilepticus (SE) is a medical emergency with significant mortality [1]. SE has been defined as continuous seizure activity, which causes neuronal cell death [2,3], epileptogenesis [3] and learning impairment [4]. Cytokines are critical mediators of specific inflammatory responses and immune reactions in the brain [5]. Tumor necrosis factor-α (TNF-α) is a 17-kDa protein that is mainly produced by activated macrophages and T cells of the immune system. TNF-α is expressed at low levels in the normal brain and is rapidly upregulated in glia, neurons and endothelial cells in various pathophysiological conditions [6]. TNF-α shows various effects on brain function depending on its local tissue concentration, the type of target cells, and especially the specific receptor subtype: TNF receptor I, or p55 receptor (TNFp55R); and TNF receptor II, or p75 receptor (TNFp75R) [7,8]. Basically, TNF-related signal transduction pathways involve NF-κB binding activity for TNFp55R contributing to cell death [9] and downstream signaling via TNFp75R involves activation of p38 mitogen-activated protein kinase to promote neuronal survival [10]. However, TNFp55R deficiency enhances KA-induced excitotoxic hippocampal injury in mice [11]. Furthermore, Marchetti et al. [12] has reported that TNFp75R-induced persistent NF-κB activity is essential for neuronal survival against excitotoxic stress. Therefore, TNF-α clearly possesses the ability to simultaneously activate both cell death and cell survival pathways, and this balance ultimately determines whether TNF-α promotes neurodegeneration or neuroprotection.

On the other hand, P2X7 receptor, a purinergic receptor, plays a role in intercellular signaling involving ATP and glutamate release. Furthermore, the release of TNF-α appears to be dependent on the P2X7 receptor. Indeed, treatment of microglia in neuron-microglia co-cultures with the P2X7 agonist 2'-3'-O-(benzoyl-benzoyl) ATP (BzATP) leads to significant reductions in glutamate-induced neuronal cell death, and either TNF-α converting enzyme inhibitor or anti-TNF-α IgG readily suppresses this protective effect [13]. In contrast, Choi et al. [14] have reported that the P2X7 receptor antagonist, oxidized ATP (OxATP), is effective in attenuating LPS-induced neuronal damage. These findings encouraged us to speculate that P2X7 receptor-mediated TNF-α regulation is involved in outcomes of SE. In the present study, therefore, we address the question of whether the effects of P2X7 receptor on the TNF-α system represent general features of SE-induced neuronal death in the hippocampus following SE.

## Methods

### Experimental animals and chemicals

This study utilized the progeny of Sprague-Dawley (SD) rats (male, 9-11 weeks old) obtained from Experimental Animal Center, Hallym University, Chunchon, South Korea. The animals were provided with a commercial diet and water *ad libitum *under controlled temperature, humidity and lighting conditions (22 ± 2°C 55 ± 5% and a 12:12 light/dark cycle with lights). Procedures involving animals and their care were conducted in accord with our institutional guidelines that comply with NIH Guide for the Care and Use of Laboratory Animals (NIH Publications No. 80-23, 1996). In addition, we have made all efforts to minimize the number of animals used and their suffering. All reagents were obtained from Sigma-Aldrich (St. Louis, MO), except as noted.

### Intracerebroventricular drug infusion

Rats were divided into eight groups, treated with either (1) saline, (2) vehicle (0.1% DMSO/saline, v/v), (3) BzATP (5 mM in saline), (4) OxATP (5 mM in saline), (5) A-438079 (10 μM in saline; Tocris Bioscience, Ellis-ville, MO), (6) A-740003 (10 μM in 0.001% DMSO/saline, v/v; Tocris Bioscience, Ellis-ville, MO), (7) soluble TNFp55R (sTNFp55R 50 μg/ml), or (8) BzATP (5 mM) + sTNFp55R (50 μg/ml). The dosage of each compound was determined as the highest dose that did not affect seizure threshold in a preliminary study. Animals were anesthetized (Zolretil, 50 mg/kg, i.m.; Virbac Laboratories) and placed in a stereotaxic frame. For osmotic pump implantation, holes were drilled through the skull to introduce a brain infusion kit 1 (Alzet, Cupertino, CA) into the right lateral ventricle (1 mm posterior; 1.5 mm lateral;--3.5 mm depth; flat skull position with bregma as reference), according to the atlas of Paxinos and Watson [15]. The infusion kit was sealed with dental cement and connected to an osmotic pump (1002, Alzet, Cupertino, CA). The pump was placed in a subcutaneous pocket in the dorsal region. Animals received 0.5 μl/hr of vehicle or compound for 2 weeks [16-18].

### Seizure induction

Three days after the start of vehicle or compound infusion, rats were treated with pilocarpine (380 mg/kg, i.p.) 20 min after methylscopolamine (5 mg/kg, i.p.). Approximately 80% of pilocarpine-treated rats showed acute behavioral features of status epilepticus (SE), including akinesia, facial automatisms, limbic seizures consisting of forelimb clonus with rearing, salivation, masticatory jaw movements, and falling. Diazepam (Valium, 10 mg/kg, i.p.; Hoffman Ia Roche, Neuilly sur-Seine) was administered 2 hours after onset of SE and repeated, as needed. The rats were then observed 3-4 hours a day in a vivarium for general behavior and occurrence of spontaneous seizures. Non-experienced SE rats (which showed only acute seizure behaviors during 10-30 min, n = 21) and age-matched normal rat were used as controls (n = 8).

### Pilocarpine-induced seizure threshold

Three days after the start of vehicle or compound infusion, some animals (n = 3) in each group were anesthetized (urethane, 1.5 g/kg, i.p.) and placed in a stereotaxic frame. Holes were drilled through the skull to introduce electrodes. The coordinates (in mm) were as follows. For the recording electrode (to the dentate gyrus):-3.8 anterior-posterior, 2.5 lateral to bregma, 2.9 depth, at a right angle to the skull surface. For the stimulating electrode (to the angular bundle): 4.2 lateral to lambda, 3.0 depth. Stainless steel electrodes (Plastics One Inc) were used for recording. Reference electrodes were placed in the posterior cranium over the cerebellum. Signals were recorded with DAM 80 differential amplifier (0.1-3000 Hz bandpass, World Precision Instruments) and data were digitized (20 kHz) and analyzed on MacChart 5 (AD Instruments). After establishing a stable baseline for at least 30 min after surgery, pilocarpine (380 mg/kg, i.p.) was given 20 min after methylscopolamine (5 mg/kg, i.p.), and latency was observed. Latency was determined as seconds from the pilocarpine injection time point to the time point showing the first seizure activity [19]. To analyze changes in EEG power value, root mean square (RMS) values were also measured.

### Tissue processing

At designated time points (Non-SE, 1 day, 2 days, 3 days and 1 week after SE, n = 5, respectively), animals were perfused transcardially with phosphate-buffered saline (PBS) followed by 4% paraformaldehyde in 0.1 M phosphate buffer (PB, pH 7.4) under urethane anesthesia (1.5 g/kg, i.p.). The brains were removed, and postfixed in the same fixative for 4 hr. The brain tissues were cryoprotected by infiltration with 30% sucrose overnight. Thereafter, the entire hippocampus was frozen and sectioned with a cryostat at 30 μm and consecutive sections were placed in six-well plates containing PBS. For stereological study, every sixth section in the series throughout the entire hippocampus was used in some animals [20].

### Immunohistochemistry

Sections were first incubated with 3% bovine serum albumin in PBS for 30 min at room temperature. Sections were then incubated in primary antibody (Table 1) in PBS containing 0.3% Triton X-100 overnight at room temperature. The sections were washed three times for 10 min with PBS, incubated sequentially, in biotinylated horse anti-mouse IgG (Vector, Burlingame, CA) and ABC complex (Vector, Burlingame, CA), diluted 1:200 in the same solution as the primary antiserum. Between incubations, the tissues were washed with PBS three times for 10 min each. The sections were visualized with 3,3'-diaminobenzidine (DAB) in 0.1 M Tris buffer and mounted on gelatin-coated slides. The immunoreactions were observed under an Axiophot microscope (Carl Zeiss, Munchen-Hallbergmoos). All images were captured using an Axiocam HRc camera and Axio Vision 3.1 software [21-23]. To identify the morphological changes induced by SE in the same hippocampal tissue, double immunofluorescent staining was also performed. Brain tissues were incubated in a mixture of goat anti-TNF-α IgG/mouse anti-calbindin D-28 k IgG (a granule cell marker) or mouse anti-GFAP IgG (an astroglial marker)/rabbit anti-TNFp55R IgG or mouse anti-GFAP IgG/TNFp75R IgG in PBS containing 0.3% triton X-100 overnight at room temperature. After washing three times for 10 minutes with PBS, sections were also incubated in a mixture of FITC-or Cy3-conjugated secondary antisera (Amersham, San Francisco, CA) for 1 hr at room temperature. Sections were mounted in Vectashield mounting media with or without DAPI (Vector, Burlingame, CA). For negative controls, rat hippocampal tissues were incubated with only the secondary antibody without primary antibody. All negative controls for immunohistochemistry resulted in the absence of immunoreactivity in any structure (data not shown).

**Table 1:** Primary Antibodies used

Antigen	Host	Manufacturer	Dilution used*
Calbindin D-28 K	rabbit	Cell signaling	1:200 (IF)

Glial fibrillary acidic protein	mouse	Millipore	1:5,000 (IF)

NeuN (a neuronal maker)	mouse	Millipore	1:1000 (IF)

NF-κB p52-Ser865	rabbit	Abcam	1:200 (IH)

NF-κB p52-Ser869	rabbit	Abcam	1:200 (IH)

NF-κB p65-Ser276	rabbit	Abcam	1:200 (IH)

NF-κB p65-Ser311	rabbit	Abcam	1:200 (IH)

NF-κB p65-Ser468	rabbit	Abcam	1:200 (IH)

NF-κB p65-Ser529	rabbit	Abcam	1:200 (IH)

TNF-α	goat	R&D system	1:500 (IH) 1:200 (IF)

TNFp55R	rabbit	Abcam	1:200 (IF)

TNFp75R	Rabbit	Abcam	1:200 (IF)

* IHC, immunohistochemistry; IF, immunofluorescence

### Fluoro-Jade B staining

Fluoro-Jade B (FJB) staining was used to identify degenerating neurons. Briefly, sections were rinsed in distilled water, and mounted onto gelatin-coated slides and then dried on a slide warmer. The slides were immersed in 100% ethanol for 3 min, followed by 70% ethanol for 2 min and distilled water for 2 min. The slides were then transferred to 0.06% potassium permanganate for 15 min and gently agitated. After rinsing in distilled water for 2 min, the slides were incubated for 30 min in 0.001% FJB (Histo-Chem Inc. Jefferson, AR), freshly prepared by adding 20 ml of a 0.01% stock FJB solution to 180 ml of 0.1% acetic acid, with gentle shaking in the dark. After rinsing for 1 min in each of three changes of distilled water, the slides were dried, dehydrated in xylene and coverslipped with DPX. For stereological study, every sixth section in the series throughout the entire hippocampus was used (see below).

### Stereology

Hippocampal volumes (V) were estimated according to a formula based on the modified Cavalieri method: V = *Σa *× *t*_nom _× 1/ssf, where *a *is area of the region of the delineated subfield measured by AxioVision Rel. 4.8 software,, *t*_nom _is the nominal section thickness (of 30 μm in this study), and ssf is the fraction of the sections sampled or section sampling fraction (of 1/6 in this study). The subfield areas were delineated with a 2.5 × objective lens. The volumes are reported as mm^3 ^[24,25]. The optical fractionator was used to estimate cell numbers. The optical fractionator (a combination of performing counting with the optical disector, with fractionator sampling) is a stereological method based on a properly designed systematic random sampling method that by definition yields unbiased estimates of population number. The sampling procedure is accomplished by focusing through the depth of the tissue (the optical disector height, *h; *of 15 μm in all cases for this study). The number of each cell type (C) in each of the subregions is estimated as: C = *ΣQ^- ^*× t/h × 1/asf × 1/ssf, where *Q^- ^*is the number of cells actually counted in the disectors that fall within the sectional profiles of the subregion seen on the sampled sections, and Asf is the area sampling fraction calculated as the area of the counting frame of the dissector, a(frame) (50 × 50 μm^2 ^in this study) and the area associated with each x, y movement, grid (x, y step) (250 × 250 μm ^2 ^in this study) {asf = [a(frame)/a(x, y step)]}. FJB-positive cells were counted with a 40 × objective lens. All FJB-positive cells were counted regardless the intensity of labeling. Cell counts were performed by two different investigators who were blind to the classification of tissues [20].

### Quantification of data

For quantification of immunohistochemical data, images were captured using an AxioImage M2 microscope and AxioVision Rel. 4.8 software (15 sections per each animal). Figures were mounted with Adobe PhotoShop v 8.0. Images were converted to gray and white images. The range of intensity values was obtained from the selected images using Adobe PhotoShop v. 8.0. Based on the mean range of intensity values, each image was normalized by adjusting the black and white range of the image using Adobe PhotoShop v. 8.0. Manipulation of the images was restricted to threshold and brightness adjustments to the whole image [21-23]. After regions were outlined, 10 areas/rat (500 μm^2^/area) were selected from the hippocampus and gray values were measured. Intensity measurements were represented as the mean number of a 256 gray scale (NIH Image 1.59 software and AxioVision Rel. 4.8 software). Values for background staining were obtained from the corpus callosum. Optical density values were then corrected by subtracting the average values of background noise obtained from 15 image inputs.

### Statistical analysis

All data obtained from the quantitative measurements and electrophysiological study were analyzed using one-way ANOVA to determine statistical significance. Bonferroni's test was used for post-hoc comparisons. A p-value below 0.05 was considered statistically significant [21-23].

## Results

### Seizure threshold

**Figure 1:**
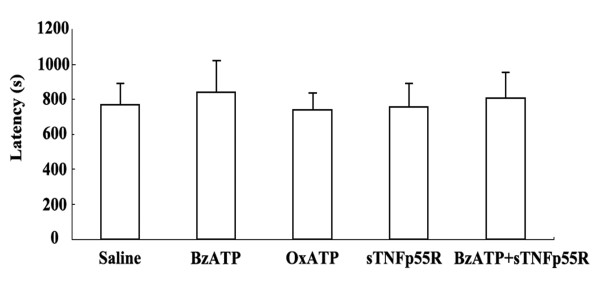
**Effects of BzATP, OxATP, sTNFp55R, and BzATP+sTNFp55R infusions on the timing of pilocarpine (PILO)-induced seizure onset**. There are no differences in seizure latency among the groups.

The criterion for time of seizure onset is the time point showing a paroxysmal depolarizing shift that is defined as lasting > 3 s and consisting of a rhythmic discharge of > 2 Hz and usually between 4 and 10 Hz. Saline-treated animals showed the beginning of epileptiform discharges 768 s after pilocarpine injection (i.p.). BzATP, OxATP, sTNFp55R and BzATP+sTNFp55R-infused animals showed the beginning of SE up to 946, 743, 763 and 816 s after pilocarpine injection, respectively, and maintenance of SE until 2 hr after SE. These findings indicate that BzATP, OxATP, sTNFp55R or BzATP+sTNFp55R-infusion did not affect pilocarpine-induced SE in rats (Figure 1).

### TNF-α expression

**Figure 2:**
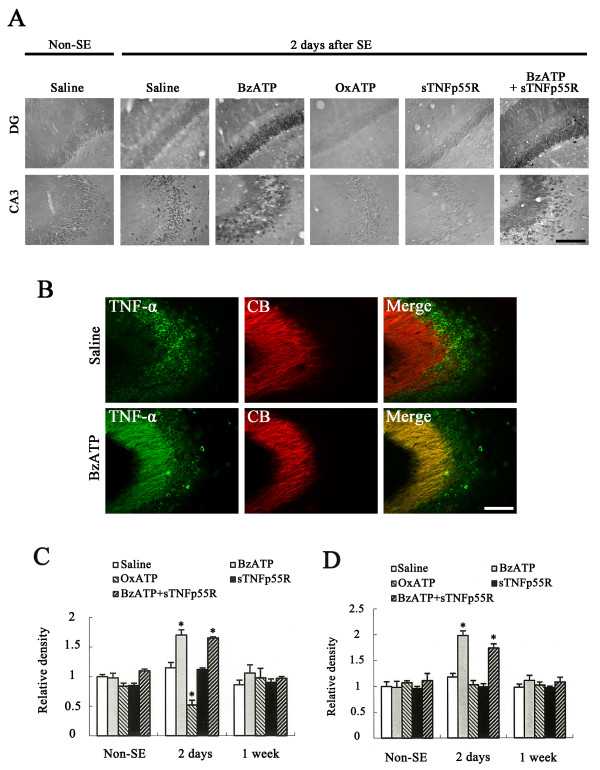
**Effects of BzATP, OxATP, sTNFp55R, and BzATP+sTNFp55R infusion on TNF-α expression following SE**. **(A) **TNF-α expression in dentate granule cells and the CA3 region 2 days after SE. Bar = 100 μm (panel 1). **(B) **TNF-α expression in mossy fibers in saline-and BzATP-infused animals 2 days after SE. In BzATP-infused animals, TNF-α immunoreactivity is colocalized with CB, a marker for mossy fibers. Bar = 100 μm (panel 1). **(C) **Quantitative analysis of TNF-α immunoreactivity in dentate granule cells following SE (mean ± S.E.M). *Value is significantly different from saline-infused animals, p < 0.05. **(D) **Quantitative analysis of TNF-α immunoreactivity in the CA3 region following SE (mean ± S.E.M). *Value is significantly different from saline-infused animals, p < 0.05.

In non-SE-induced animals of saline-infused groups, TNF-α immunoreactivity was weakly detected in CA1-3 pyramidal cells and dentate granule cells. In addition, hilar neurons also showed TNF-α immunoreactivity (Figure 2A). This localization pattern of TNF-α immunoreactivity in the hippocampus was consistent with previous studies [26-29]. BzATP-, OxATP-or sTNFp55R-infusion did not affect the localization pattern of TNF-α immunoreactivity in the hippocampus (data not shown). Two days after SE, TNF-α immunoreactivity was slightly increased (not statistically significant) in the hippocampus of saline-infused animals, as compared to non-SE-induced animals (Figures 2A-B). In BzATP-infused animals, TNF-α immunoreactivity in dentate granule cells was significantly increased 1.7-fold as compared with that in saline-infused animals (p < 0.05; Figures 2A-C). In addition, TNF-α immunoreactivity was readily apparent in the mossy fibers (stratum lucidum), while TNF-α immunoreactivity in CA1-3 pyramidal cells was unaltered (p < 0.05; Figures 2A-B and 2D). In OxATP-infused animals, TNF-α immunoreactivity in dentate granule cells was significantly decreased to about 50% that in saline-infused animals (p < 0.05; Figures 2A and 2C). In A-438079-or A-740003-infused animals, alterations in TNF-α immunoreactivity in dentate granule cells were similar to those in OxATP-infused animals (data not shown). In sTNFp55R-and BzATP+sTNFp55R-infused animals, the alterations in TNF-α immunoreactivity were similar those observed in saline-and BzATP-infused animals, respectively (Figures 2A and 2C). One week after SE, TNF-α immunoreactivity in the hippocampus recovered to the level of non-SE-induced animals within every group (Figures 2C-D). TNF-α immunoreactivity was also detected in microglia 1-7 days after SE (data not shown). However, there was no difference in TNF-α immunoreactivity within microglial cells of each group.

### TNFp55R expression

**Figure 3:**
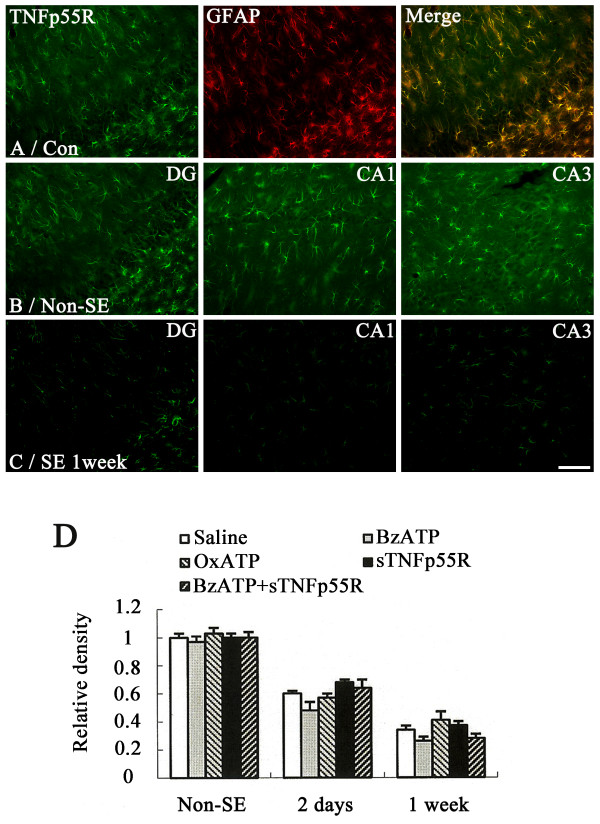
**Effect of SE on TNFp55R expression**. **(A) **Astroglial expression of TNFp55R in control animal. Bar = 100 μm. **(B) **Distribution of TNFp55R immunoreactivity in hippocampus of a non-SE-induced animal in the saline-infused group. Bar = 100 μm. **(C) **Distribution of TNFp55R immunoreactivity in hippocampus of a 1-week post-SE animal in the saline-infused group. Bar = 100 μm. **(D) **Quantitative analysis of TNFp55R immunoreactivity in hippocampus following SE (mean ± S.E.M). There are no differences in TNFp55R immunoreactivity in hippocampus among the groups.

In non-SE-induced animals of saline-infused groups, TNFp55R immunoreactivity was observed mainly in GFAP-positive astrocytes (Figure 3A). Similarly, BzATP-, OxATP-, A-438079, A-740003 or sTNFp55R-infusion did not affect the localization pattern of TNF-α immunoreactivity in the hippocampus of non-SE-induced animals (data not shown). As compared to non-SE-induced animals (Figure 3B), TNFp55R immunoreactivity was gradually reduced in astrocytes 1-7 days after SE (P < 0.05, Figures 3C-D). BzATP, OxATP, A-438079, A-740003, sTNFp55R or BzATP+sTNFp55R infusion did not affect changes in TNFp55R immunoreactivity in the hippocampus following SE (data not shown).

### TNFp75R expression

**Figure 4:**
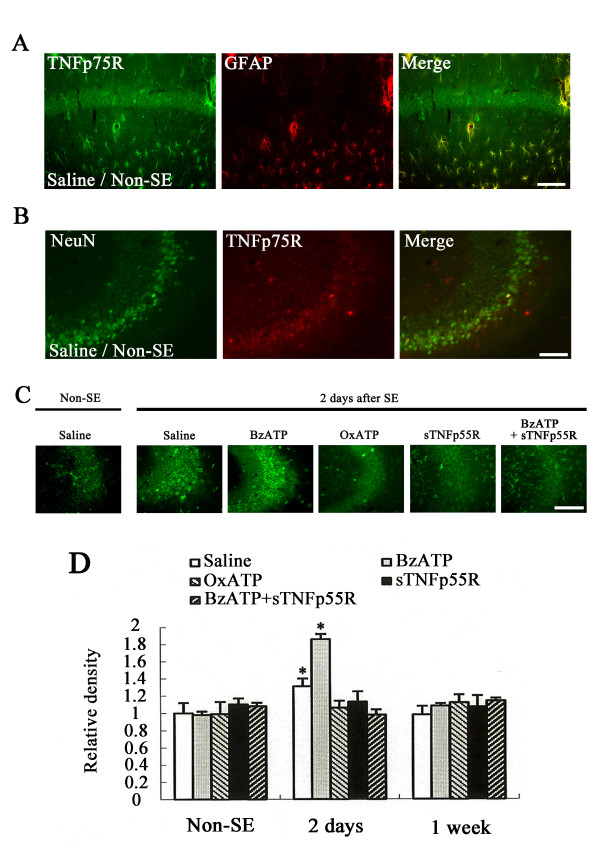
**Effects of BzATP, OxATP, sTNFp55R, and BzATP+sTNFp55R infusions on TNFp75R expression following SE**. **(A) **Double immunofluorescence for TNFp75R and GFAP in CA1 region of a non-SE-induced animal in the saline-infused group. Bar = 100 μm. **(B) **Double immunofluorescence for TNFp75R and NeuN in CA3 region of a non-SE-induced animal in the saline-infused group. Bar = 100 μm. **(C) **TNFp75R expression in the CA3 region 2 days after SE Bar = 100 μm (panel 1). **(D) **Quantitative analysis of TNFp75R immunoreactivity in the CA3 region following SE (mean ± S.E.M). *Value is significantly different from saline-infused animals, p < 0.05.

In non-SE-induced animals of saline-infused groups, TNFp75R immunoreactivity was observed in neurons and GFAP-positive astrocytes (Figures 4A-B). BzATP, OxATP, A-438079, A-740003, sTNFp55R or BzATP+sTNFp55R infusion did not affect changes in TNFp55R immunoreactivity in the hippocampus (data not shown). Two to three days after SE, TNFp75R immunoreactivity was increased, exclusively in CA3 neurons, to 1.3-(2 days after SE) and 1.5-(3 days after SE, data not shown) fold in saline-infused animals, as compared with that in non-SE-induced animals (P < 0.05, Figures 4C-D). In BzATP-infused animals, TNFp75R immunoreactivity was increased, only in CA3 neurons, 1.7-(2 days after SE) and 1.8-(3 days after SE, data not shown) fold as compared with that in saline-infused animals (P < 0.05, Figures 4C-D). OxATP, sTNFp55R or BzATP+sTNFp55R infusions effectively prevented changes in TNFp75R immunoreactivity in the CA3 region following SE (Figures 4C-D). The effect of A-438079-or A-740003 infusion on TNFp75R immunoreactivity was similar to that of OxATP infusion (data not shown). One week after SE, TNFp75R immunoreactivity in the CA3 region recovered to the levels of non-SE-induced animals within every group (Figure 4D).

### Neuronal damage

**Figure 5:**
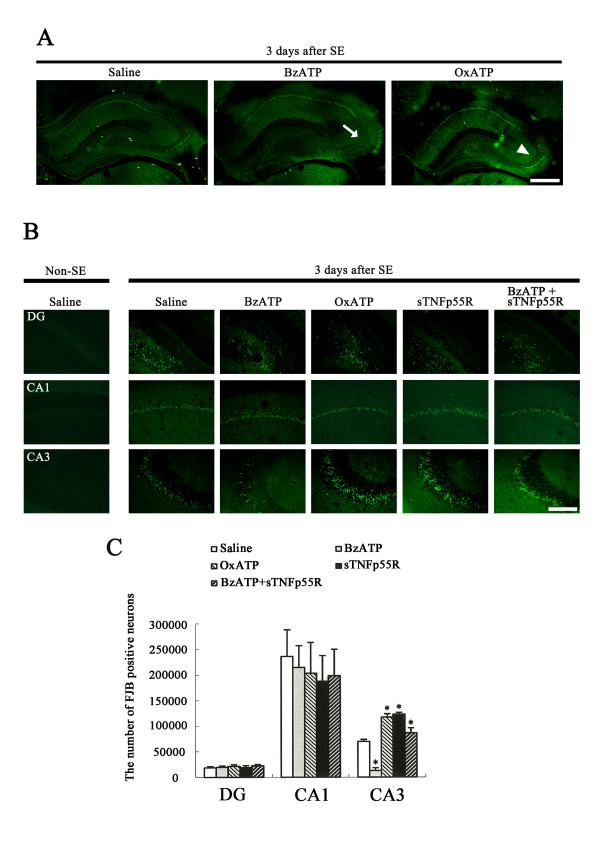
**Effect of BzATP, OxATP, sTNFp55R, and BzATP+sTNFp55R infusions on SE-induced neuronal death**. **(A) **Representative photographs of FJB staining following SE. As compared to saline-infusion, BzATP infusion attenuates neuronal damage in the CA3 region (arrows), while OxATP infusion worsens it (arrowheads). **(B) **SE-induced neuronal damages in dentate gyrus, and in the CA1 and CA3 regions 3 days after SE. Bar = 100 μm. **(C) **Quantitative analysis of neuronal damage in dentate gyrus, and in the CA1 and CA3 regions 3 days after SE (mean ± S.E.M). BzATP infusion alleviates SE-induced neuronal damage only in the CA3 region. However, the other treatments increase SE-induced neuronal damage. *Value is significantly different from saline-infused animals, p < 0.05.

In our previous study [30] and in preliminary studies here, neuronal damage was first detectable 3 days after SE. Therefore, we applied FJB stains to 3-day post-SE animals of each group. Few FJB positive neurons were detected in the hippocampus of non-SE-induced animals in any group (data not shown). In saline-infused animals, FJB-positive neurons were detected in CA1-3 pyramidal cells and dentate hilus neurons (Figures 5A-C). The number of FJB-positive neurons in dentate gyrus, CA1 and CA3 regions was 18,215 ± 2,568, 236,145 ± 51,976 and 69,469 ± 4,367, respectively (Figures 5B-C). For BzATP-infused animals, the number of FJB-positive neurons in dentate gyrus, CA1 and CA3 regions was 19,138 ± 2,841, 214,843 ± 42,368 and 12,418 ± 5,714, respectively (Figure 5A). Thus, BzATP infusion attenuated SE-induced neuronal damage in the CA3 region (P < 0.05, Figures 5B-C). In contrast, OxATP-, A-438079, A-740003, sTNFp55R and BzATP+sTNFp55R infusion increased the number of FJB-positive neurons in the CA3 region to 117,428 ± 6,468, 131,456 ± 5,196, 129,345 ± 7,138, 122,987 ± 3,568 and 86,468 ± 9,789, respectively (Figures 5B-C). However, OxATP-, sTNFp55R or BzATP+sTNFp55R infusion could not exacerbate SE-induced neuronal damages in dentate gyrus or the CA1 region, as compared to BzATP-infusion (Figures 5B-C).

### NF-κB phosphorylation

**Figure 6:**
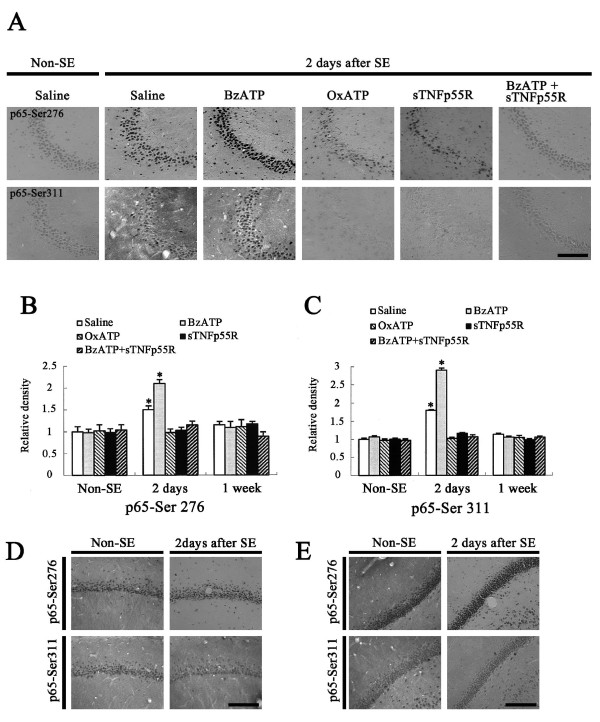
**Effect of BzATP, OxATP, sTNFp55R, and BzATP+sTNFp55R infusions on SE-induced NF-κB phosphorylation**. **(A) **p65-Ser276 and p65-Ser311 phosphorylation in the CA3 region following SE. Bar = 100 μm. **(B) **Quantitative analysis of p65-Ser276 phosphorylation in CA3 region following SE (mean ± S.E.M). *Value is significantly different from saline-infused animals, p < 0.05. **(C) **Quantitative analysis of p65-Ser311 phosphorylation in CA3 region following SE (mean ± S.E.M). *Value is significantly different from saline-infused animals, p < 0.05. (**D-E**) p65-Ser276 and p65-Ser311 phosphorylations in CA1 and in dentate granule cells following SE. As compared to non-SE animals, there is no difference in p65-Ser276 or p65-Ser311 phosphorylations in these regions 2 days after SE. Bar = 100 μm.

It is well established that TNF-α is a major stimulus to phosphorylation of NF-κB. To confirm TNF-α-mediated signaling following SE, we performed an immunohistochemical study using six phospho-NF-κB antibodies. As compared to control animals, p52-Ser865, p52-Ser869, p65-Ser468, and p65-Ser529 NF-κB phosphorylations were unaltered in nuclei of CA1 and CA3 pyramidal cells, dentate granule cells, and hilar neurons 2 days after SE (data not shown). However, both p65-Ser276 and p65-Ser311 phosphorylations were increased, only in the CA3 region, following SE. In non-SE-induced animals of saline-infused groups, moderate p65-Ser276 immunoreactivity was observed in nuclei of CA3 neurons (Figures 6A-B). p65-Ser311 immunoreactivity was also weakly detected in nuclei of CA3 neurons (Figures 6A-B and 6C). BzATP-, OxATP-, A-438079, A-740003, or sTNFp55R-infusion did not affect the localization patterns of p65-Ser276 or p65-Ser311 immunoreactivity in the hippocampus of non-SE-induced animals (data not shown). Two days after SE, both p65-Ser276 and p65-Ser311 immunoreactivities in CA3 neurons were enhanced to 1.5-and 1.8-fold in saline-infused animals, respectively (Figures 6A-C). In BzATP-infused animals, both p65-Ser276 and p65-Ser311 immunoreactivities in CA3 neurons were increased 2.1-and 2.9-fold as compared with that in non-SE-induced animals (Figures 6A-C). OxATP-, A-438079, A-740003, sTNFp55R-or BzATP+sTNFp55R infusions effectively prevented increases in p65-Ser276 and p65-Ser311 immunoreactivities in CA3 neurons following SE (Figures 6A-C). One week after SE, phospho-NF-κB immunoreactivities were decreased to non-SE-induced animal levels (Figures 6B-C). Following SE, however, both p65-Ser276 and p65-Ser311 immunoreactivities were unaltered in CA1 pyramidal cells (Figure 6D) as well as dentate granule cells (Figure 6E). Furthermore, BzATP-, OxATP-, A-438079, A-740003, or sTNFp55R infusions did not affect the localization pattern of p65-Ser276 immunoreactivity in these regions following SE (data not shown).

## Discussion

It is well established that normal rat brain constitutive expresses biologically active TNF-*α *as well as TNF-*α *mRNA [31-33] and that TNF-*α *may be produced by neurons themselves [33]. These previous studies reveal that TNF-*α *may serve as a mediator of neurotransmitter release in the CNS. The P2X7 receptor is identified as a mediator in response to acute brain injury, since the synthesis and membrane localization of P2X7 receptor are rapidly up-regulated in response to various stimuli, including SE [31,34-37]. The P2X7 receptor engages diverse signal cascades, which include initiation of rapid release and processing of proinflammatory cytokines including TNF-*α *[34,36,38]. Similar to previous studies [26-29], the present study shows that TNF-α immunoreactivity is readily apparent in hippocampal neurons as well as dentate granule cells in non-SE-induced animals. Interestingly, BzATP-infusion increased TNF-α immunoreactivity in dentate granule cells following SE, while OxATP-infusion decreased it. These findings indicate that P2X7 receptor-mediated regulation of TNF-α expression may not be a consequence of distinct effects of each drug on seizure activity. This is because BzATP-, OxATP-, A-438079, A-740003, sTNFp55R-and BzATP+sTNFp55R infusions did not affect pilocarpine-induced SE in rats. Furthermore, these infusions could not affect basal level of TNF-α immunoreactivity in the hippocampus. Therefore, the present findings suggest that alterations in SE-induced TNF-α immunoreactivity may be mediated by P2X7 receptor function.

TNF-α clearly possesses the ability to simultaneously activate both cell death and cell survival pathways, and this balance ultimately determines whether TNF-α promotes neurodegeneration or neuroprotection. Basically, TNF-related signal transduction pathways include NF-κB binding activity for TNFp55R contributing to cell death [9] and downstream signaling via the TNFp75R involves activation of p38 mitogen-activated protein kinase to promote neuronal survival [10]. In the present study, BzATP-infusion caused a restricted increase in TNF-α immunoreactivity within dentate granule cells and their axons, and mossy fibers, following SE. BzATP-infusion also enhanced TNFp75R expression in response to TNF-α overexpression, only in CA3 neurons, which synapse with mossy fibers. Furthermore, the present study shows that BzATP infusion attenuates SE-induced neuronal damage, only in the CA3 region, while OxATP-, A-438079, A-740003, sTNFp55R and BzATP+sTNFp55R infusions exacerbate neuronal damage as compared to saline-infused animals. Therefore, our findings suggest that TNF-α-mediated signaling may play a neuroprotective role against SE.

It has been reported that p65-Ser276 and p65-Ser311 phosphorylations of NF-κB induced by TNF-α enhance their transactivation potentials and their interactions with cAMP response element-binding (CREB)-binding protein (CBP), which is also important for the survival of neurons [39-44]. In the present study, BzATP-infusion enhanced TNFp75R expression with intensification of p65-Ser276 and p65-Ser311 immunoreactivities following SE. In addition, sTNFp55R pretreatment could not prevent SE-induced neuronal damages, and BzATP+sTNFp55R infusion did not show protective effect of BzATP. These findings indicate that the activation of TNFp75R may protect CA3 neurons from SE via p65-Ser276 and p65-Ser311 NF-κB phosphorylations.

Microglia are a major producer of TNF-α in brain [45,46]. Hide et al. [26] reported that TNF-α release from microglia is induced by BzATP. P2X7 receptor expression is increased in the rat hippocampus following pilocarpine-induced SE [30,47]. With respect to these previous reports, it is likely that TNF-α released from microglia may also play a neuroprotective role in the rat hippocampus following SE. In the present study, however, there was no difference in TNF-α immunoreactivity between microglia of each group. Although the present data could not provide biological mechanism of this phenomenon, it may be considered that the dosages of OxATP, A-438079 and A-740003 applied in the present study are insufficient to inhibit TNF-α expression in microglia due to full P2X7 receptor expression. Further studies are needed to elucidate the effectiveness of P2X7 receptor agonists and antagonists to alter TNF-α expression in activated microglia.

In conclusion, the present study suggests that TNF-α induction by P2X7 receptor activation may ameliorate SE-induced CA3 neuronal damage via enhancement of p65-Ser276 and p65-Ser311 phosphorylations of NF-κB.

## Competing interests

The authors declare that they have no competing interests.

## Authors' contributions

JEK was involved in designing and performing all experiments. HJR, TCK helped in drafting the manuscript. JEK and HJR did the immunohistochemistry, the intracerebroventricular drug infusion, the seizure studies and the acquisition of data and analyses. TCK provided continuous intellectual input, and evaluation and interpretation of data. All authors read and approved the final manuscript.
